# Sequential generation of linear cluster states from a single photon emitter

**DOI:** 10.1038/s41467-020-19341-4

**Published:** 2020-10-30

**Authors:** D. Istrati, Y. Pilnyak, J. C. Loredo, C. Antón, N. Somaschi, P. Hilaire, H. Ollivier, M. Esmann, L. Cohen, L. Vidro, C. Millet, A. Lemaître, I. Sagnes, A. Harouri, L. Lanco, P. Senellart, H. S. Eisenberg

**Affiliations:** 1 Racah Institute of Physics, Hebrew University of Jerusalem, 91904 Jerusalem, Israel; 2 CNRS Centre for Nanoscience and Nanotechnology, Université Paris-Sud, Université Paris-Saclay, Palaiseau, France; 3 Quandela, Palaiseau, France; 4 Université Paris Diderot, Paris, France

**Keywords:** Quantum optics, Quantum information, Single photons and quantum effects

## Abstract

Light states composed of multiple entangled photons—such as cluster states—are essential for developing and scaling-up quantum computing networks. Photonic cluster states can be obtained from single-photon sources and entangling gates, but so far this has only been done with probabilistic sources constrained to intrinsically low efficiencies, and an increasing hardware overhead. Here, we report the resource-efficient generation of polarization-encoded, individually-addressable photons in linear cluster states occupying a single spatial mode. We employ a single entangling-gate in a fiber loop configuration to sequentially entangle an ever-growing stream of photons originating from the currently most efficient single-photon source technology—a semiconductor quantum dot. With this apparatus, we demonstrate the generation of linear cluster states up to four photons in a single-mode fiber. The reported architecture can be programmed for linear-cluster states of any number of photons, that are required for photonic one-way quantum computing schemes.

## Abstract (web summary)

Generating photonic cluster states using a single non-heralded source and a single entangling gate would optimise scalability and reduce resource overhead. Here, the authors generate up to 4-photon cluster states using a quantum dot coupled to a fibre loop, with a fourfold generation rate of 10 Hz.

## Introduction

Optical quantum technologies include a wide range of applications, from quantum sensing^1,2^, to quantum communication^3^, and computing^4–6^. Entanglement is the most common resource for these applications^7^, exploiting various degrees-of-freedom, e.g., polarization, time-frequency, orbital angular momentum, and spatial modes. The generation of high quality large cluster states can be used for photonic one-way quantum computing^5,6,8^, which is favorable compared to the widely used Turing-like gate-based model in solid-state systems^4^. Moreover, photonic cluster states have been proposed to implement measurement-based quantum communication networks^9–11^, an architecture that promises long distance quantum communication at higher rates compared to other memory-based counterparts.

Cluster states are a special class of multipartite graph states that show improved robustness to loss compared to GHZ-states, or W-states^12–14^. They can be generated using single-photon sources and entangling operations. So far, they have been implemented using probabilistic nonlinear sources of photon pairs up to six photons, by the *χ*^(2)^ parametric downconversion (PDC), and the *χ*^(3)^ four-wave mixing processes, or, for continuous variable encoding, by multimode squeezing in optical parametric oscillators^6,15–19^. Photonic GHZ states of up to twelve entangled photons have also been obtained in this manner^20,21^. The common approach consists of multiple sources and multiple well balanced paths to manipulate the photons. In this approach, it is important to operate in a regime where the probability to generate a pair is low (typically few percents) in order to limit multipair emission. These low source efficiencies make the protocols difficult to scale up to large photon numbers, in addition to an increased resource budget when employing multiplexed schemes^22,23^. A more scalable way to produce large photonic cluster states has been proposed in 2009^24^, making use of a single quantum emitter embedding a spin acting as a quantum memory. A proof-of-concept experimental demonstration has been provided with a semiconductor quantum dot up to two photons^25^. However, this was obtained at very low generation rates, and with considerable challenges to allow further scalability, such as the need for longer spin coherence times and efficient polarization-independent photon extraction.

In this work, we demonstrate an approach for the generation of photonic cluster states that takes advantage of an already proven and available technology for single-photon generation—semiconductor quantum dots (QDs)^26^—and a recent proposal for entanglement generation based on temporal delay-loops^27^. Quantum dots generate single-photons on demand with near-unity indistinguishability^28–33^, and high single-photon purity. In addition, they can have high in-fiber brightness (defined as the probability to have a single-photon coupled into a single-mode fiber per excitation pulse), typically one order-of-magnitude larger than heralded single-photon sources^28,29^. This allows for an exponential increase in multiphoton generation rates, which has already been used for Boson sampling^34–36^, and on-chip quantum walks^37^. We employ a fiber delay-loop apparatus to sequentially entangle photons successively generated by a bright QD single-photon source. Our experimental demonstration brings the record for the number of entangled photons from a single emitter from two photons^32,38,39^ to four photons. Linear photonic cluster states of two, three, and four photons are obtained, with a four photon generation rate of  ~10 Hz, assuming perfect detection efficiency. Our compact entangling apparatus allows for both entanglement generation and polarization state analysis. Additionally, we define a parameter, the scaling-ratio, to quantify prospects of scalability, and to allow comparison between different implementations.

## Results

### Experimental scheme

**Fig. 1:**
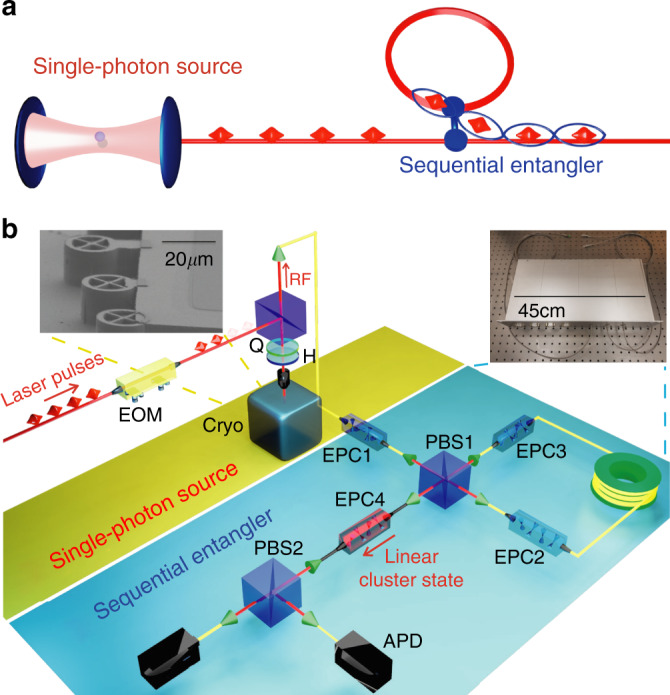
A fibered source of linear photonic cluster states. **a** Working principle of the source: single-photons generated in successive time bins, seperated by 74 ns, are sent into an apparatus where a delay-loop stores a photon until it meets with the next one at the entangling gate. **b** Top inserts: the physical implementation. A single InGaAs quantum dot photon source in an electrically-connected cavity, and an all-fibered entangling optical circuit in a 19'' box. Bottom: Detailed experimental setup—EOM electro-optic modulator, Cryo cryostat, Q quarter-wave plate, H half-wave plate, RF resonance fluorescence, EPC electrically-driven polarization controller, PBS polarizing beam-splitter, APD avalanche photon detectors; see text for additional details.

  Figure 1a presents the principle of the proposed scheme. A single quantum dot positioned in an optical cavity serves as an efficient single-photon source. Periodical excitation with optical pulses leads to the emission of a stream of single-photons. The separated emission times enable individual addressability of each photon. The single-photons are then sent into an entangling gate, where the time between emissions is tuned to match the length of a delay-loop, that serves as a quantum memory. As a result, the combined system constitutes a source of linear cluster states encoded in the polarization degree-of-freedom, and individually addressable in the time domain. With this protocol, linear cluster states of any length can be produced, controlled by the number of consecutive photons sent into the entangling apparatus. Figure 1a depicts the case for four photons. All entangling operations occur at the same entangling gate, hence the low resource requirements of our approach.

Figure 1b depicts the physical implementation of our protocol. The single-photon sources used here are made of a single InGaAs quantum dot deterministically positioned in an electrically connected pillar cavity^28,40^ with optical resonances around 925 nm. The electrically controlled emitter-cavity coupling ensures efficient collection of photons through accelerated spontaneous emission into the cavity mode. The QD transition is coherently controlled with resonant excitation pulses from a Ti:Sapphire laser operating at 81 MHz repetition rate. Maximum source efficiency is achieved by setting the excitation at *π*-pulse level, and the single photons are collected in a crossed polarization scheme. In this work, we used several sources with various characteristics: sources based on either neutral or charged excitons, with in-fiber brightness between 4 and 15%. The detected single-photon rate out-of-fiber varies from 0.8 to 3 MHz using standard silicon avalanche photon detectors (APD) with 25% detection efficiency typically. The degree of indistinguishability *M* for photons generated by a single source at various time delays was measured for all sources, and varies from 0.77 to 0.95, the higher values being obtained with spectral filtering to remove contribution from phonon sidebands (see Supplementary Note 6). The laser pump driving the single-photon source is modulated by an electro-optic intensity modulator (EOM), that creates the required sequence for entangling the desired number of photons.

The entangling apparatus is implemented in an all-fibered compact device, packaged into a standard 19" rack mountable box, see inset in Fig. 1b. It has one single-mode fiber input, a fiber delay-loop about 15 m long, and one "fusion" gate^41^ implemented by a polarizing beam-splitter (PBS). The output of this first PBS1 is sent to a single-mode fiber from where the entangled photons emerge. The analysis setup is also included in the same box. It consists of another projecting PBS2, whose two fiber outputs exit the box. Four electrically-driven polarization controllers, labeled EPCi, contain four voltage-controlled birefringent elements each. The birefringence is induced by mechanically squeezing the fiber. The squeezing axes are oriented at 45° relative to each other. Thus, this configuration allows full control over the photons’ polarization. EPC1 is positioned before the delay loop, and used to align the source photon polarization. The next two polarization controllers EPC2 and EPC3, are inside the loop. EPC2 compensates for the fiber arbitrary polarization rotation between PBS1 and the alignment of the first element of EPC3. As the incoming photon into this first element is perfectly aligned along the ordinary birefringent axis, it only experiences a temporal phase shift rather than a polarization rotation. This is used as a phase scanning mechanism in our setup, described later on. After applying the phase, the other three elements of EPC3 are set to compensate for the arbitrary rotation of the fiber between EPC3 and PBS1. EPC4 is positioned after the delay loop, and is used to align the photon polarization at the output of PBS1 and the input of PBS2. At the output of PBS2, two single-photon detectors are temporally synchronized to the laser clock frequency, and the time analysis of the state is controlled using a custom-designed FPGA controller. The required temporal synchronization is between the repetition rate of the laser used to excite the source, the generated pulse sequence of the EOM that selects the input photon pulses, and the detection events. Once this is achieved between two photons, any larger number of injected photons will also be synchronized as they all experience the same loop delay time (see Supplementary Note 1).

### Entangling protocol

The sequential operation of the entangling apparatus can be described in the following manner. The excitation laser is switched on and off using the EOM to create a series of consecutive laser pulses, corresponding to the temporal sequence of single-photons to be entangled. Each modulated pump sequence is preceded by two empty cycles in order to ensure that the delay loop is empty at the beginning of the protocol. The generated single-photons are injected into the sequential entangler, see Fig. 1b, and their state is set to the diagonal polarization relative to PBS1 orientation $\left|p\right\rangle =\frac{1}{\sqrt{2}}(\left|h\right\rangle +\left|v\right\rangle )$ via EPC1, where $\left|h\right\rangle$ and $\left|v\right\rangle$ designate the horizontal and vertical polarization states, respectively. Similarly, the antidiagonal state is defined as $\left|m\right\rangle =\frac{1}{\sqrt{2}}(\left|h\right\rangle -\left|v\right\rangle )$. After the first photon leaves PBS1, it is in a spatial superposition of being transmitted as the $\left|h\right\rangle$ (reflected as the $\left|v\right\rangle$) state. Consequently, the protocol succeeds if the first photon enters the loop, verified by post selection of not detecting a photon at the first time-bin. Inside the loop, EPC2 compensates for arbitrary polarization rotations, and EPC3 controls the birefringent phase *φ*, and rotates the $\left|h\right\rangle$ state of EPC2 orientation to the $\left|p\right\rangle$ state of PBS1. Each photon is delayed for *τ* ≃ 74 ns, corresponding to six laser cycles. When the photon inside the loop arrives to PBS1, it is timed to entangle with a new photon from the single photon source. This is achieved by fine adjustment of the pump laser repetition rate.

Postselecting each photon to exit from a different port of the entangling PBS1, the two diagonal photons are projected onto the maximally entangled state:

1 $$\left|p\right\rangle \otimes \left|p\right\rangle \mathop{\longrightarrow }\limits_{{\mathrm{selection}}}^{{\mathrm{post}}}\left|{\phi }^{+}\right\rangle =\frac{1}{\sqrt{2}}\left(\left|{h}_{1}{h}_{2}\right\rangle +\left|{v}_{1}{v}_{2}\right\rangle \right),$$

where the subscripts 1, 2 refer to the photon detection times *τ*, 2*τ*. Photon 1 has left the loop towards the detectors. Photon 2 remains in the loop, where it is rotated by EPC3 to the *p*/*m* polarization basis, resulting in the state $\frac{1}{\sqrt{2}}\left(\left|{h}_{1}{p}_{2}\right\rangle +\left|{v}_{1}{m}_{2}\right\rangle \right)$, which is a two-photon linear cluster in graph representation^42^. The conditional detection of photon 1 at time *τ* and photon 2 at time 2*τ* or later, verifies the postselection condition.

When a third photon enters the setup, it arrives at the entangling PBS1 at the $\left|p\right\rangle$ state. The outcome of the entangling PBS1 is:

2 $$\frac{1}{\sqrt{2}}\left(\left|{h}_{1}{p}_{2}\right\rangle +\left|{v}_{1}{m}_{2}\right\rangle \right)\otimes \left|p\right\rangle \to \frac{1}{\sqrt{2}}\left(\left|{h}_{1}{\phi }_{2,3}^{+}\right\rangle +\left|{v}_{1}{\phi }_{2,3}^{-}\right\rangle \right)\ .$$

Thus, the new photon is entangled with the two previous photons into a GHZ state $\frac{1}{\sqrt{2}}\left(\left|{p}_{1}{h}_{2}{p}_{3}\right\rangle +\left|{m}_{1}{v}_{2}{m}_{3}\right\rangle \right)$, where the photon remaining in the loop (now photon 3) is rotated by EPC3. This three-photon GHZ state is a linear cluster in graph representation. When a fourth photon enters the setup, repeating the above protocol, the resulting entangled state is not a GHZ state, but the four-photon linear cluster (LC) state:

3 $$\left|{\psi }_{{\rm{LC}}}^{(4)}\right\rangle = \, \frac{1}{2}\left(\left|{p}_{1}{h}_{2}{h}_{3}{p}_{4}\right\rangle +\left|{p}_{1}{h}_{2}{v}_{3}{m}_{4}\right\rangle \right.\\ \,\,\left.+\left|{m}_{1}{v}_{2}{h}_{3}{p}_{4}\right\rangle -\left|{m}_{1}{v}_{2}{v}_{3}{m}_{4}\right\rangle \right).$$

The conditional detection of a photon at each of the first *n* − 1 time slots starting at *τ* and on, as well as detecting the last *n*th photon at time *nτ* or later, verifies the post-selection condition for all the projections.

**Fig. 2:**
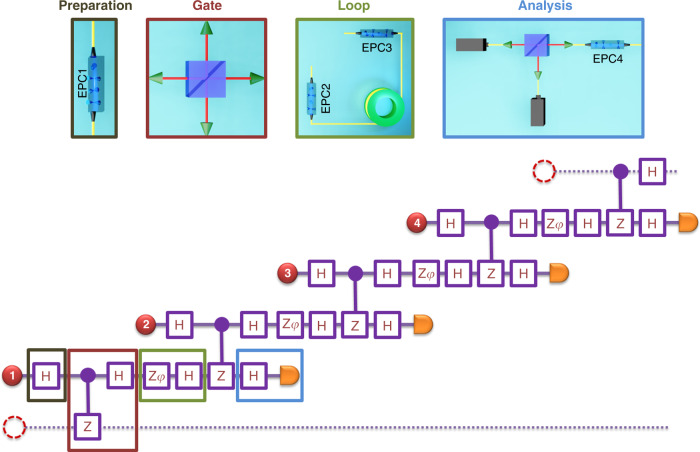
Quantum circuit representation of the entangling scheme. The table maps the used logical operations, the Hadamard *H* and *Z*_*φ*_ transforms and the controlled-phase gate with their corresponding physical elements from Fig. 1. First, each temporally separated photon (red disks) undergoes a state preparation step at EPC1 (black box) before entering the entangling gate (red box) and the delay loop with EPC2 and EPC3 (green box). When exiting the source, the photons reach the analysis step (blue box) of EPC4 and the photon polarization sensitive detection. Thus, the independent four successive incoming photons are transformed into a four-photon cluster state. The dashed circles/lines depict the absent photons right before and after the injected photon sequence.

Local unitary operations on each photon may transform $\left|{\psi }_{{\rm{LC}}}^{(4)}\right\rangle$ to other equivalent graph states^42^. When more photons come in a timely manner, they are entangled into an ever-growing linear cluster state^27^. Figure 2 depicts the quantum logic circuit implementation corresponding to this protocol.

### Quantum state analysis

In our protocol, correlations are detected by photon measurements at consecutive time slots. The polarization analysis procedure is performed by applying *X* or *Z* Pauli operators by EPC4 and PBS2 to the first *n* − 1 photons. The last *n*th photon inside the loop is projected on PBS1 (the entangling PBS). If projected onto the $\left|h\right\rangle$ polarization, it exits the loop, and if projected onto $\left|v\right\rangle$, it stays inside for another cycle. Thus, the last photon polarization is analyzed by its arrival time to either detector.

The projection of *n* photons results in 2^n^ possible amplitudes. In order to demonstrate the quantum nonlocality of the produced states, the *n*-photon amplitudes are interfered^43,44^. This nonlocal interference is achieved by rotating the measurement basis of the first *n* − 1 photons to the diagonal basis via a Hadamard rotation. In this case, half of the photons’ probability amplitudes interfere constructively while the other half interfere destructively. The difference between these two amplitude groups is indicative of the level of quantum interference. This level can be quantified by ${V}_{n}={\rm{Tr}}\left({X}^{\otimes n}\hat{\rho }\right)$, where $\hat{\rho }$ is the density matrix of the generated *n*-photon state. In order to accumulate more information about the nonlocal interference, the phase *φ* is applied by the operator ${Z}_{\varphi }^{\otimes (n-1)}\otimes I$ to the *n* − 1 photons, where *I* is the unit operator and ${Z}_{\varphi }=I\cos (\varphi /2)-iZ\sin (\varphi /2)$. When this phase is scanned, the different amplitude probabilities, and thus also *V*_n_ oscillate, revealing more information about the degree of entanglement (see Supplementary Note 2). The maximal value of *V*_n_(*φ*) is used to evaluate the quality of the generated state.

**Fig. 3:**
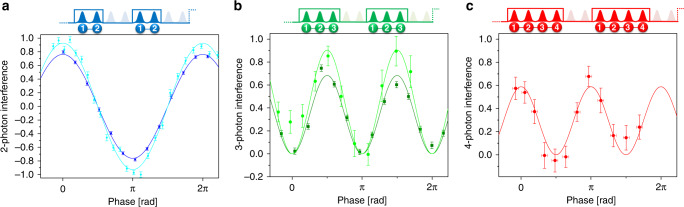
Quantum interference of two, three, and four photon cluster states. The corresponding EOM pulse sequences are depicted above the graphs. **a** Phase scan of *V*_2_ for a two-photon state with a fitted maximum of 0.76 ± 0.01 at 480 Hz, 30 s per data point (dark blue), and 0.93 ± 0.02 at 50 Hz, 6 s per data point (light blue). **b** Phase scan of *V*_3_ for a three-photon state with a fitted maximum of 0.68 ± 0.03 at 4.3 Hz, 11 min per data point (dark green), and 0.90 ± 0.07 at 0.33 Hz, 8 min per data point (light green). **c** Phase scan of ${V}_{4^{\prime} }$ for a four-photon state with a fitted maximum of 0.59 ± 0.04 at 0.04 Hz, 50 min per data point. Error bars are calculated assuming Poisson distribution. Phase error bars for **c** are calculated from two-photon residuals fit for multiple scans. Background counts from residual unfiltered pump and detector after-pulsing events are subtracted (see Supplementary Note 7).

  Figure 3 shows the resulting phase-dependent oscillations in *V*_n_, for the cases of two, three and four photons. Assuming that the main source for imperfect interference is the two-photon indistinguishability *M*, the predicted two-photon, and three-photon quantum interference levels are ${V}_{2}(\varphi )=M\cos (\varphi )$, and ${V}_{3}(\varphi )\,=\,{M}^{2}\frac{1\,-\,\cos (2\varphi )}{2}$. The corresponding four-photon value *V*_4_ has an upper theoretical limit of $\frac{2}{3\sqrt{3}}\,\simeq\,0.38$, which limits the experimental sensitivity. Therefore, we present ${V}_{4^{\prime} }={\rm{Tr}}\left(X\otimes I\otimes X\otimes X\hat{\rho }\right)$ which can reach 1, as this observable is part of the stabilizer group of $\left|{\psi }_{{\rm{LC}}}^{(4)}\right\rangle$ before the rotation of the last photon (see Supplementary Note 3). The dependence of this four-photon interference level on *φ* is ${V}_{4^{\prime} }(\varphi )\,=\,{M}^{2}\frac{1+\cos (2\varphi )}{2}$ (see Supplementary Note 4). Figure 3c shows our experimental results for ${V}_{4^{\prime} }$. The measurements presented in Fig. 3 were obtained with two QD sources. The first one, corresponding to a positively charged QD operated with a 10 pm spectral filtering to reduce the contribution of phonon sideband emission, showed a resulting in-fiber brightness of about 4%, and *M* = 0.95 ± 0.01. It allows observing high interference levels for two and three photons. The other one corresponds to a negatively charged QD with an in-fiber brightness of about 15% but a reduced indistinguishability of *M* = 0.77 ± 0.01. It allows implementing the scheme up to four photons within a measurement time of 10 h for the complete data set. Although the indistinguishability was below the typical one observed with these QD sources^45^, this source still allows the generation of four-photon cluster states with an improved scalability, as discussed later on.

**Fig. 4:**
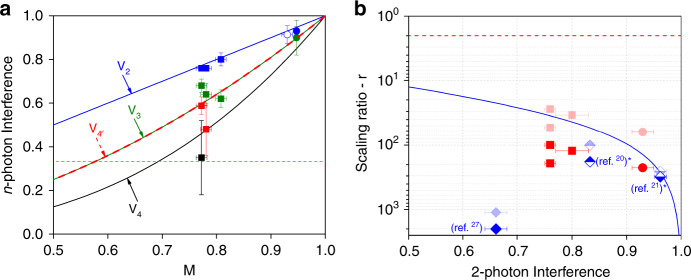
Entanglement criteria. In both panels, solid squares and solid circles represent results using a charged excitation, with and without spectral filtering, respectively, while the empty circle represents results for neutral excitation without spectral filtering. **a** *n*-photon interference vs. indistinguishability values *M* of various experiments. Blue, green, red, and black represent *V*_2_, *V*_3_, ${V}_{4^{\prime} }$, and *V*_4_ results, respectively. The curves represent calculated theoretical values for noise-induced distinguishability. Results for *V*_4_ are normalized to one. Horizontal dashed magenta line is the entanglement length interference threshold. **b** A comparison between scaling ratios as a function of the two-photon interference for various single-photon source implementations. Solid blue diamond correspond to an experiment implemented with a PDC source and a free space setup^27^. Half-filled blue diamonds correspond to the calculated value, where the best PDC source currently available^20,21^ would have been used in our setup. Red symbols correspond to the results of the present work. Light-color symbols represent calculated values for *η*_d_ = 0.9. (*) The scaling ratios for the refs. ^20,21^ were calculated based on published photon rates, interpreted as an heralding single-photon source. Dashed red (solid blue) line represents the theoretical probabilistic gate (heralded PDC sources) limit. See text for further details.

We have repeated the measurements using several of the mentioned sources based on negatively charged, positively charged or neutral dots, the latter two showing higher *M*. For some measurements, the indistinguishability was further increased using an etalon filter, but at the cost of a reduced count rate. In Fig. 4a, the quantum interference values of these measurements are presented for various values of *M*, obtained by standard Hong-Ou-Mandel (HOM) interference^46,47^, and the *g*^(2)^(0) value from second-order intensity correlations^48^ (see Supplementary Note 6). The solid lines represent the theoretical expected *n*-photon interference levels, showing good agreement with experiment.

It is possible to define a bounded witness measure ${\mathcal{W}}\,\le\,\tilde{{\mathcal{W}}}$^49^ that detects entanglement of cluster states when $\langle \tilde{{\mathcal{W}}}\rangle\,<\,0$. This inequality would also be satisfied for equivalent states up to a local unitary operation, but those equivalent states require large transformations to the cluster state and are highly improbable in our experiment (see Supplementary Note 5). From the experimental results presented in Fig. 3 we obtain the values for the two-photon, three-photon, four-photon measurements: $\langle {\tilde{{\mathcal{W}}}}_{2}\rangle =-0.32\pm 0.01$, $\langle {\tilde{{\mathcal{W}}}}_{3}\rangle =-0.22\pm 0.01$, $\langle {\tilde{{\mathcal{W}}}}_{4}\rangle =-0.04\pm 0.02$ certifying the generation of cluster states. In addition, the results from the more indistinguishable source are $\langle {\tilde{{\mathcal{W}}}}_{2}\rangle =-0.45\pm 0.02$, $\langle {\tilde{{\mathcal{W}}}}_{3}\rangle\,=\,-0.41\,\pm\,0.02$. All are negative and imply the generated entanglement. Furthermore, as the state fidelity is related to its witness as ${\mathcal{F}}=\frac{1}{2}-{\mathcal{W}}$, all of the quoted values imply a better than 50% fidelity.

Imperfect photon indistinguishability *M* limits the entanglement length, defined as the longest possible linear cluster state such that positive concurrence is found between the first and last photons of the chain when all the others are measured^27^. This length is an upper limit for how far can quantum information flow along the linear state during the one-way quantum computation procedure^5^. We find that for an *n*-photon entanglement length, the threshold interference value is ${V}_{n}=\frac{1}{3}$, independent of *n*. All the presented results are above this threshold. Moreover, our results correspond to maximum entanglement lengths between 23 and 5 photons (see Supplementary Note 8). In addition to this criterion, the three-photon genuine entanglement can be evaluated by the *V*_3_ measure, as the three-photon cluster state matches a GHZ state. The interference threshold value for a three-photon GHZ state is $\frac{1}{2}$^50^, which is well exceeded by all our measurements, with values ranging from *V*_3_ = 0.62 ± 0.04 to *V*_3_ = 0.90 ± 0.08, depending on the source used (see Fig. 4a).

### Scaling ratio

It is instructive to quantify and compare between the scalability prospects of our scheme using a QD source and those of heralded PDC sources. To this end, we define the scaling ratio *r*—the reduction factor of detection rates when one photon is added to the protocol. The smaller the scaling ratio, the better the scalability, where the ultimate goal is to reach the ideal value of *r* = 1, enabling the deterministic entangling of any number of photons. In our demonstration, the entangling operation has a 50% chance to succeed, thus this setup can only reach a value of *r* = 2. The detection rate for *n*-photon events is:

4 $${R}_{n}=R{\left({\eta }_{{\rm{d}}}{\eta }_{{\rm{s}}}{\eta }_{{\rm{l}}}{\eta }_{{\rm{b}}}\right)}^{n}{\eta }_{{\rm{g}}}^{n-1},$$

where *R* is the single-photon repetition rate, and the *η*’s represent various system efficiencies. Most efficiencies apply to every photon, such as the detector efficiency *η*_d_, the system loss without the delay loop *η*_s_, the delay loop (memory cycle) loss *η*_l_, and the source brightness *η*_b_, including both its quantum yield and overall optical collection efficiency into a single-mode fiber including spectral filtering. One other efficiency doesn’t apply to the first photon, the entangling gate efficiency *η*_g_. Thus, the scaling ratio is:

5 $$r={R}_{n}/{R}_{n+1}={\left({\eta }_{{\rm{d}}}{\eta }_{{\rm{s}}}{\eta }_{{\rm{b}}}{\eta }_{{\rm{g}}}{\eta }_{{\rm{l}}}\right)}^{-1}.$$

Figure 4b presents this scaling ratio as a function of the two-photon interference level for various theoretical and experimental situations. The use of a probabilistic gate with *η*_g_ = 0.5 limits the scaling ratio to *r* ≥ 2 (dashed red line) considering that *η*_d_ = *η*_s_ = *η*_b_ = *η*_l_ = 1. An intrinsic limitation arises when operating the present scheme with heralded PDC sources. For such sources, the two-photon interference reduces when increasing the source efficiency. The solid blue line represents the dependence of the scaling ratio on the two-photon interference level $r\,=\,2\frac{1\,+\,{V}_{2}}{1\,-\,{V}_{2}}$, considering *η*_d_ = *η*_s_ = *η*_l_ = 1 and *η*_g_ = 0.5 (see Supplementary Note 9). It represents an intrinsic upper limit for PDC sources, a limit that could only be overcome by multiplexing schemes^23^, yet at the cost of increasingly demanding resources and reduced single-photon repetition rate *R*.

The symbols in Fig. 4b present a scaling ratio values for various implementations. The solid blue symbols correspond to an equivalent scheme but implemented with a PDC source and a free space setup^27^. The half-filled blue symbols correspond to the predicted values where the same experimental scheme would be implemented using the best pulsed PDC source currently available^20,21^. The red symbols correspond to the present work, with measured efficiency values of *η*_d_ = 0.25, *η*_s_ = 0.7, *η*_l_ = 0.75, 0.04 ≤ *η*_b_ ≤ 0.15 depending on the source used. The light-blue and light-red data points extrapolate previous implementations, predicted ones and the present experimental results to the case *η*_d_ = 0.9, since such detection efficiencies are currently available at all considered wavelegnths by replacing the silicon APDs with superconducting nanowire single-photon detectors^51^. This allows for a better comparison between different implementations.

The presented comparison shows that our current results, obtained with a lossy and imperfect setup, already approaches the upper limit of lossless PDC sources (see Supplementary Note 9). Further improved scaling ratios are thus expected to be within reach with QD sources in the near future. The QD source brightness can be increased by a factor of up to 2 by changing the excitation schemes following recent propositions^52,53^. The fibered brightness could also be increased by using a larger numerical aperture collection lens and engineering a better mode matching with the single mode fiber. Moreover, the setup efficiencies *η*_l_ and *η*_s_ could also be improved by reducing losses arising mostly from imperfect fiber coupled PBSs, increasing efficiencies to *η*_s_ = *η*_l_ = 0.9. In addition, fast polarization elements could double the generation rate by ensuring that the first photon always enters the loop. The cluster state generation rate including all of these improvements will increase by about 2 × 11^n^ relative to our current results. Without changing the pump repetition rate, a 10-photon event per 30 s and a 12-photon event per 40 min are expected, presenting a scaling ratio of *r* = 9.

Using the scaling ratio, a comparison between our approach to the generation of cluster states from a source of entangled photon pairs^6,15^ is not possible. Such schemes use setups that can only generate states of a fixed number of photons, and have to be physically extended in order to accommodate for more. Nevertheless, there are other suggested methods that can be examined using the scaling ratio parameter. Using ancillary photons and linear optical elements have good prospects for scalability, as it can achieve deterministic photon entanglement. However, the deterministic generation of a four-photon cluster state with the most efficient scheme, requires 26 entangled photon pairs^41^, thus becoming advantageous only for scaling ratio values *r* < 1.06 (see Supplementary Note 10). A better scaling ratio than *r* = 2 is also possible when using a spin–photon interface to remove the probabilistic operation of the entangling gate^24^. Such approach remains very challenging experimentally: a first implementation reached a scaling ratio of 700 for a two-qubit fidelity of 0.73 ± 0.06^25^. As long as our method includes the 50% postselection bottleneck, its scaling ratio is limited to *r* ≥ 2. Nevertheless, it does not require entangled photon sources nor ancillary photons nor spin–photon interaction.

## Discussion

In conclusion, we have reported the generation of multiphoton linear cluster states using a single quantum emitter coupled to a compact entangling-loop configuration. The measurement of nonlocal quantum interference demonstrates genuine multiparticle entanglement up to four photons. As our protocol relies only on a single quantum emitter and a single entangling gate, the scheme can provide the best possible scalability ratios using linear optics. The present experimental demonstration, although using both imperfect quantum dot sources and entangling apparatus, already demonstrates a scaling ratio on a par with the best possible level predicted for heralded PDC single-photon sources. Straightforward technical improvements, both on the source operation side and on the setup design, will allow reaching larger photon numbers in the near future. Additional delay loops could be used to generate cluster states of higher dimensionality. Finally, removing the last bottleneck of the 50% probabilistic entangling operation is also foreseeable, considering recent progress in engineering of photon–photon interactions using natural or artificial atoms^54–57^. Thus, the present multiphoton entanglement scheme promises a path for scaling up quantum computation and communication protocols^58–60^.

## Supplementary information

Supplementary Information

## Data Availability

The data that support the findings of this study are available from the corresponding author upon reasonable request.
