# Supplementary material for: Sequential generation of linear cluster states from a single photon emitter
Source: Nat Commun. 2020 Oct 30;11:5501. doi: 10.1038/s41467-020-19341-4 (PMC7603328; doi:10.1038/s41467-020-19341-4)
Supplement: Supplementary file 1 — Supplementary Information [file 41467_2020_19341_MOESM1_ESM.pdf]

Supplementary Information for:
Sequential generation of linear cluster states from a single photon emitter

Istrati and Pilnyak et al.

SUPPLEMENTARY NOTE 1 - EXPERIMENTAL PREPARATION AND MEASUREMENT PROCEDURE

The cluster generation setup uses four electrically driven polarization controllers (EPCs), allowing the transformation of any given input polarization to any desired output polarization of a single photon. Their method of operation uses a local magnetically-induced transverse mechanical stress on the fiber. Each such EPC integrates four channels, where for each channel, the orientation of the applied stress is rotated by an angle of 45 degrees, relative to the neighboring channel. This local change to the fiber refractive index applies any birefringent phase in the range up to 2π, inducing a polarization rotation. Depending on the polarization of the incoming photon, a single channel can be used as a retardation element whereby it can serve as a phase inducing mechanism between different photons of orthogonal polarizations.

The key factor in deciding the fiber loop length, equivalent to a temporal separation of 74 ns, is overcoming the nominal dead-time of the single-photon detectors ($\sim$60 ns). This fiber loop length is equivalent to 6 consecutive laser pulses, under a standard repetition rate of $\sim$81 MHz. Thus, in this cluster generation scheme, each photon generated at the ith time slot was interfered with a photon generated 74 ns later, generated at the ith+6 time slot.

For an n-photon experiment, a train of laser pulses separated by 12.3 ns, used for the excitation of the emitter, is converted by an electro-optical modulator (EOM) into a 74 ns time-separated sequence of n pulses. For example, in the generation of a two-photon cluster state, the excitation sequence consists of an *on-on-off-off* EOM modulation pattern, or equivalently denoted in binary form as 1100. For each *on*(*off*) bin, the EOM transmits(blocks) the laser pulses for a duration of $\sim$74 ns. The *off* periods are necessary to reduce the probability of a remaining photon inside the loop before the start of the next sequence. Therefore, the whole temporal duration of the sequence is 296 ns, where each *on* period is comprised of six laser pulses. In this case, a total of 12 photons are injected into the fiber setup, allowing the fiber setup to interfere photons 1-7, 2-8, etc. For the three- (four-) photon experiment, a 11100 (111100) sequence is applied with the EOM, interfering photons in time slots 1-7-13, 2-8-14, etc. (1-7-13-19, 2-8-14-20, etc.).

A comprehensive custom Field-Programmable Gate Array (FPGA) digital electronics hardware was developed for the entire control over the generation of electrical signals for the EOM and data acquisition from the single photon detectors. This system was also implemented with the facility to time delay all electrical signals (input and output) as well as the synchronized collection of detection events into specific time-bin counters. All this was integrated directly into the FPGA fabric. In addition, a custom developed computer software was programmed to collect, analyze and real-time display all the experimental data, as well as control high-current digital to analog converters (DAC) for the correct operation of all EPCs.

The single photon detector signal was collected by the FPGA board into time-bins, thereby assigning time tags to detection events. The 0th time-bin denoted the case where a single photon was emitted, did not enter the fiber-loop and immediately triggered a detection event. This is essentially the fastest route by which an emitted photon may be detected. Detection events at any other time-bin, assuming a single photon was emitted, mean that the photon was delayed inside the loop. In this case, the time-bin's index denotes the number of loop iterations experienced by the photon.

SUPPLEMENTARY NOTE 2 - PHASE CHANNEL ALIGNMENT

A natural approach to demonstrate the nonlocal interference would be to set the measurement basis of the photons to $\frac{1}{\sqrt{2}}\left(|h\rangle \pm e^{i\theta_i}|v\rangle\right)$ and change the delay time between pulses. Yet, it would require changing the repetition rate of the laser. Instead, we set the repetition rate to perfectly match the length of the delay loop, and control a birefringent phase φ between the different populations of an n-photon entangled state. This phase is added before the polarization rotation inside the delay loop. The polarization controllers EPC1, EPC3 and EPC4 (see Fig. 1 of the main text) perform the rotations for θ_1, θ_2 and θ_3, respectively. For the two-photon case, after the PBS projection and post-selection, the following state is produced:

$$|\phi\rangle = \frac{1}{\sqrt{2}}\left(|h_1h_2\rangle + e^{i(\theta_1+\theta_2)}|v_1v_2\rangle\right), \tag{1}$$

The addition of a phase between the states results in:

$$|\phi\rangle = \frac{1}{\sqrt{2}}\left(|h_1h_2\rangle + e^{i(\theta_1+\theta_2+\varphi)}|v_1v_2\rangle\right). \tag{2}$$

The next step consists on rotating the polarization of the photon exiting the loop into the basis $\frac{1}{\sqrt{2}}\left(|h\rangle \pm e^{i\theta_3}|v\rangle\right)$, and rotating the photon remaining in the loop into the basis $\frac{1}{\sqrt{2}}\left(|h\rangle \pm e^{i\theta_2}|v\rangle\right)$. An interference measurement in this

case would exhibit a dependence on φ.

In order to create a controllable phase channel in the fiber system, we align EPC2 and EPC3, where channel 1 in EPC3 (EPC3_1) acts as the phase channel of φ

$$\mathrm{Z}_{\varphi} = \begin{bmatrix} e^{-i\varphi/2} & 0 \\ 0 & e^{i\varphi/2} \end{bmatrix} \tag{3}$$

SUPPLEMENTARY NOTE 3 - QUANTUM INTERFERENCE OBSERVABLE FROM THE STABILIZER GROUP

Cluster states can be defined by the appropriate set of stabilizer group [1]. These groups are defined by combinations of the Pauli operators I, X, Y, Z. Each stabilizer group comprises the eigenbasis for a specific cluster state.

In our experiment, we describe the produced states before the transformation performed by EPC3 and EPC4. In case of using fast active elements with this scheme, any unitary operation could be performed on any photon. Here, the operation on photons 1 to $n-1$ is the same while the operation on the nth photon is X, as it is the configuration for the entangling gate of all photons. This imposes the requirement for the observable to be used.

For the two-qubit cluster state

$$|\phi^+\rangle = \frac{1}{\sqrt{2}}\left(|h_1h_2\rangle + |v_1v_2\rangle\right), \tag{4}$$

the corresponding stabilizers are $I{\otimes}I$, $X{\otimes}X$, $Z{\otimes}Z$, and $-Y{\otimes}Y$. Thus, for a measurement of $|\phi^+\rangle$ with the stabilizer $X{\otimes}X$ we expect an eigenvalue 1. The outcome measurement, as changing the state with respect to the phase φ, is detailed in the next section.

For the three-qubit state

$$|\psi\rangle = \frac{1}{2}\Big[|(h+v)_1h_2h_3\rangle + |(h-v)_1v_2v_3\rangle\Big], \tag{5}$$

$X{\otimes}X{\otimes}X$ is not a part of the stabilizer group and the projection measurement would return zero. However, applying the phase φ described by the operator $Z_{\varphi}^{\otimes 2}{\otimes}I$, setting $\varphi{=}\frac{\pi}{2}$ we get the state

$$|\psi\rangle = \frac{1}{2}\Big[|(h+iv)_1h_2h_3\rangle + i|(h-iv)_1v_2v_3\rangle\Big], \tag{6}$$

for which $X{\otimes}X{\otimes}X$ is part of the stabilizer group and thus the eigenvalue is 1.

The four-photon cluster state produced in this experiment

$$\begin{aligned}|\psi^4\rangle = \frac{1}{2^{\frac{3}{2}}}\Big[&|(h+v)_1h_2h_3h_4\rangle + |(h+v)_1h_2v_3v_4\rangle \\ &+|(h-v)_1v_2h_3h_4\rangle - |(h-v)_1v_2v_3v_4\rangle\Big],\end{aligned} \tag{7}$$

is the eigenvector of the stabilizer group with the generators (up to a Hadamard unitary transformation on the last photon these are the generators of the four-qubit linear cluster state) $g_1{=}X{\otimes}Z{\otimes}I{\otimes}I$, $g_2{=}Z{\otimes}X{\otimes}Z{\otimes}I$, $g_3{=}I{\otimes}Z{\otimes}X{\otimes}X$, $g_4{=}I{\otimes}I{\otimes}Z{\otimes}Z$.

We identify that operating the parity operator $X^{\otimes 4}$ would not give an eigenvalue of 1 as it is not a stabilizer of the state. However, we can identify the operator $g1{\cdot}g3{=}X{\otimes}I{\otimes}X{\otimes}X$ as part of this group ensuring $|\psi^4\rangle$ is an eigenvector. This operator could be measured in the experimental setup without fast active elements. In the next section we derive the behaviour of this when $|\psi^4\rangle$ is changed with φ.

As our scheme enlarges the state in the form of a cluster state with added photons, we can derive a similar observable, as for $n = 4$, to any even number of photons. This is deduced from the form of the stabilizer group. We look at the generators of an n-qubit cluster (up to a Hadamard unitary transformation on the last photon). The first generators are $g_1{=}X{\otimes}Z{\otimes}I{\otimes}I{\otimes}I_{n-4}$, $g_2{=}Z{\otimes}X{\otimes}Z{\otimes}I{\otimes}I_{n-4}$, $g_3{=}I{\otimes}Z{\otimes}X{\otimes}Z{\otimes}I_{n-4}$, for $2{<}k{<}n{-}1$ the generator would be $g_k{=}I_{k-2}{\otimes}Z{\otimes}X{\otimes}Z{\otimes}I_{n-k-1}$, while the last two generators are $g_{n-1}{=}I_{n-4}{\otimes}I{\otimes}Z{\otimes}X{\otimes}X$, $g_n{=}I_{n-4}{\otimes}I{\otimes}I{\otimes}Z{\otimes}Z$, where $I_m{=}I^{\otimes m}$. Multiplication of generators gives rise to the stabilizer group. From certain multiplications between the generators, we find the stabilizer

$$S_{V_{n'}} = \prod_{i=1,3,..}^{n-1} g_i = (X \otimes I)^{\otimes\left(\frac{n}{2}-1\right)} \otimes X \otimes X, \tag{8}$$

for which the n-qubit state is an eigenvector.

SUPPLEMENTARY NOTE 4 - QUANTUM INTERFERENCE LEVEL CALCULATION

The presented interference levels are calculated from the appropriate projection of the n-photon state as described in the main text. Each projection results in 2^n population terms. The general state

$$|\psi_\varphi^n\rangle = \sum_{k=1}^{2^n} a_{\psi_k}(\varphi)|\psi_k\rangle, \tag{9}$$

where $|\psi_k\rangle$ are the 2^n states that span the n-qubit space in the h/v basis.

For the two-photon state

$$|\psi\rangle = \frac{1}{\sqrt{2}}\left(|h_1 h_2\rangle + e^{i\varphi}|v_1 v_2\rangle\right). \tag{10}$$

measuring along the $X{\otimes}X$ basis gives 4 amplitudes

$$\begin{aligned} a_{\text{hh}} = a_{\text{vv}} &= \frac{1}{2^{\frac{3}{2}}}(1+e^{i\varphi}) \\ a_{\text{hv}} = a_{\text{vh}} &= \frac{1}{2^{\frac{3}{2}}}(1-e^{i\varphi}) \end{aligned} \tag{11}$$

From this, the phase dependent two-photon quantum interference level is calculated

$$V_2 = P_{\text{hh}} - P_{\text{hv}} - P_{\text{vh}} + P_{\text{vv}} = \cos(\varphi), \tag{12}$$

where P_{ψ_k}=$|a_{\psi_k}|^2$.

In a similar way the three-photon state

$$|\psi\rangle = \frac{1}{2}\Big[|(h+e^{i\varphi}v)_1 h_2 h_3\rangle + e^{i\varphi}|(h-e^{i\varphi}v)_1 v_2 v_3\rangle\Big]. \tag{13}$$

Measuring along $X^{\otimes 3}$ gives 8 amplitudes

$$\begin{aligned} a_{\text{hhh}} = a_{\text{hvv}} &= \frac{1}{2^{\frac{5}{2}}}(1+2e^{i\varphi}-e^{i2\varphi}) \\ a_{\text{hhv}} = a_{\text{hvh}} = a_{\text{vhh}} = a_{\text{vvv}} &= \frac{1}{2^{\frac{5}{2}}}(1+e^{i2\varphi}) \\ a_{\text{vhv}} = a_{\text{vvh}} &= \frac{1}{2^{\frac{5}{2}}}(1-2e^{i\varphi}-e^{i2\varphi}). \end{aligned} \tag{14}$$

From this, the phase dependent three-photon quantum interference level is calculated

$$\begin{aligned} V_3 &= P_{\text{hhh}} - P_{\text{hhv}} - P_{\text{hvh}} + P_{\text{hvv}} \\ &\quad - P_{\text{vhh}} + P_{\text{vhv}} + P_{\text{vvh}} - P_{\text{vvv}} \\ &= \frac{1-\cos(2\varphi)}{2} = \sin^2(\varphi). \end{aligned} \tag{15}$$

The four-photon state is

$$\begin{aligned} |\psi_\varphi^4\rangle = \frac{1}{2^{\frac{3}{2}}}\Big[&|(h+e^{i\varphi}v)_1 h_2 h_3 h_4\rangle + e^{i\varphi}|(h+e^{i\varphi}v)_1 h_2 v_3 v_4\rangle \\ &+e^{i\varphi}\left(|(h-e^{i\varphi}v)_1 v_2 h_3 h_4\rangle - e^{i\varphi}|(h-e^{i\varphi}v)_1 v_2 v_3 v_4\rangle\right)\Big]. \end{aligned} \tag{16}$$

Measuring along $X^{\otimes 4}$ gives 16 amplitudes

$$\begin{aligned} a_{\text{hhhh}} = a_{\text{hhvv}} = a_{\text{vhhv}} = a_{\text{vhvh}} &= \frac{1}{2^{\frac{7}{2}}}(1+3e^{i\varphi}-e^{i2\varphi}+e^{i3\varphi}) \\ a_{\text{hhhv}} = a_{\text{hhvh}} = a_{\text{vhhh}} = a_{\text{vhvv}} &= \frac{1}{2^{\frac{7}{2}}}(1-e^{i\varphi})(1+e^{i\varphi})^2 \\ a_{\text{hvhv}} = a_{\text{hvvh}} = a_{\text{vvhh}} = a_{\text{vvvv}} &= \frac{1}{2^{\frac{7}{2}}}(1+e^{i\varphi})(1-e^{i\varphi})^2 \\ a_{\text{hvhh}} = a_{\text{hvvv}} = a_{\text{vvhv}} = a_{\text{vvvh}} &= \frac{1}{2^{\frac{7}{2}}}(1+3e^{i\varphi}+e^{i2\varphi}-e^{i3\varphi}) \end{aligned} \tag{17}$$

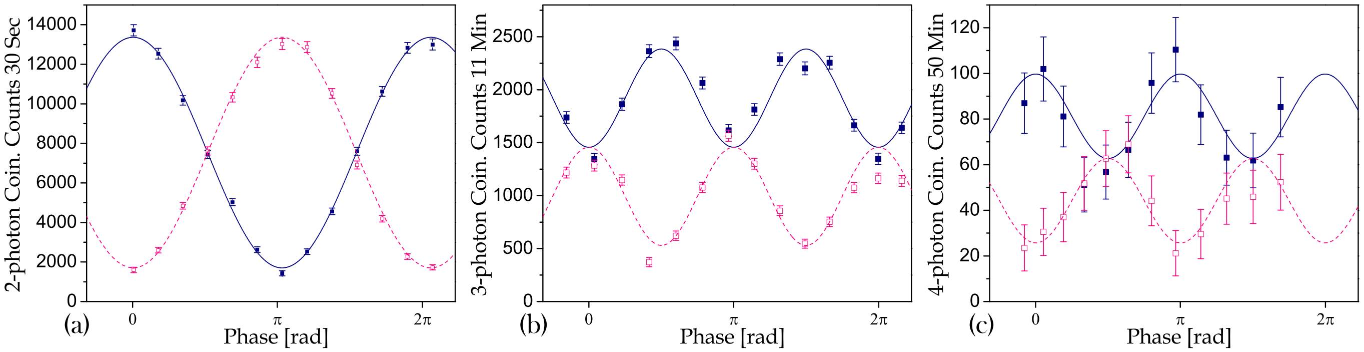

Supplementary Figure 1. Coincidence counts for the sum of the population terms for (a) two-photon (b) three-photon (c) four-photon measurements. From these results the quantum interference plots in Fig. 3 in the main text were calculated.

The experimental results of two, three, and four-photon measurements, from which the coincidence counts of the sum of the populations with constructive and destructive interference are calculated, is represented in Supplementary Figure 1. From this, the level of interference is calculated by dividing the difference of the constructive and destructive interference by their sum. Consequently, the four-photon quantum interference level, for the observable $S_{V_{4'}}=X\otimes I\otimes X\otimes X$ defined in Supplementary Eq. 8 is calculated

$$V_{4'} = Tr(S_{V_{4'}}\hat{\rho}) = P_1 - P_2 - P_3 + P_4 = \frac{1+\cos(2\varphi)}{2} = \cos^2(\varphi)\,, \tag{18}$$

while for the observable $X^{\otimes 4}$

$$V_4 = \mathrm{Tr}(X^{\otimes 4}\hat{\rho}) = P_1 - P_2 + P_3 - P_4 = \frac{-\cos(\varphi)+\cos(3\varphi)}{4} = -\cos(\varphi)\sin^2(\varphi)\,, \tag{19}$$

the maximum this observable can obtain is $\frac{2}{3\sqrt{3}}$. This interference level from the measured results with a π phase is presented in Supplementary Figure 2.

As the entanglement process of our scheme is a recurring process, in the linear cluster picture it is simply adding another photon and link to the chain, thus we are able to deduce a quantum interference level measure to any n-photon state ($n \geq 3$)

$$V_n = \mathrm{Tr}(X^{\otimes n}\hat{\rho}) = -\cos^{n-3}(\varphi)\sin^2(\varphi)\,. \tag{20}$$

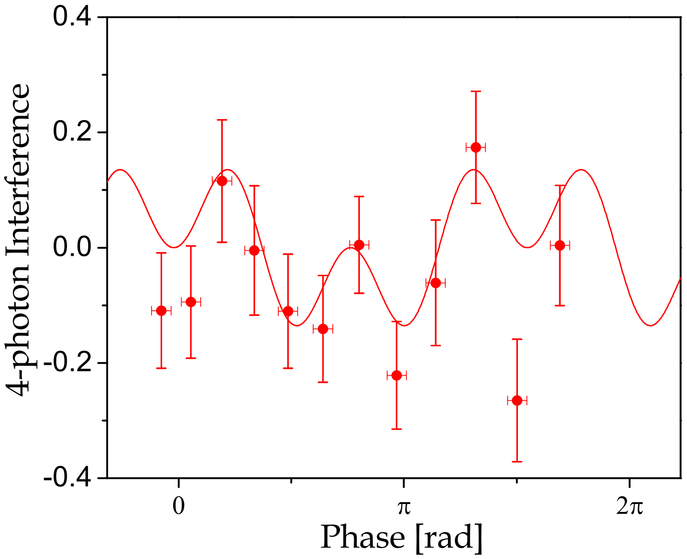

Supplementary Figure 2. The normalized interference level $V_4 = 0.35 \pm 0.17$.

This observable is not part of the stabilizer group of the produced state at $\varphi = 0$. In addition, when varying φ the maximum amplitude is less than 1 for $n > 3$. In a similar way we may deduce the n-photon quantum interference level to the observable defined in Supplementary Eq. 8. This observable is valid to an even n-photon state, as it is part of its stabilizer group, giving unity at $\varphi = 0$

$$V_{n'} = \mathrm{Tr}(S_{V_{n'}}\hat{\rho}) = \cos^{\frac{n}{2}}(\varphi)\,. \tag{21}$$

SUPPLEMENTARY NOTE 5 - BOUNDED ENTANGLEMENT WITNESS

In order to evaluate the level of entanglement of an experimental result, it is desirable to measure the states at multiple experimental settings in order to evaluate the expectation value of the witness operator $\mathcal{W}$. As our setup is indeed configurable, but with a limited reconfiguration speed, most degrees of freedom of the individual photons are inaccessible. Thus, we have to adopt a few assumptions on the generated state in order to bound the expectation value of its entanglement witness such that $\langle\tilde{\mathcal{W}}\rangle\geq\langle\mathcal{W}\rangle$, for which $\langle\tilde{\mathcal{W}}\rangle<0$ certifies the presence of entanglement [2].

Here, we will use two such assumptions. Firstly, as all photons are processed through the same optical path and entangled at the same PBS element, we consider the quality of any interference measurement that involves a certain number of photons to represent the level of similar interference of any permutation of the same photon number. As more photons are involved, the interference level decreases, as it is affected more by imperfections.

The second assumption considers that the generated state is not far from a linear cluster state, up to local Pauli operations. This assumption is based on our knowledge of the generation setup and uncertainties in its alignment.

In addition, due to good agreement between the interference level of two, three, and four photons with the noise model of two-photon indistinguishability presented in Supplementary Eq. 29 (see Fig. 4a in the main text), expectation values that involve X and Y observables bound expectation values with Z and I. This is a common result as dephasing effects are more probable than full depolarization.

With these assumptions and result, it is possible to bound the entanglement witness expectation value using the quantum interference amplitudes. The two-photon state witness bound is $\langle\tilde{\mathcal{W}}_2\rangle=\frac{1}{2}-\frac{1}{4}\mathrm{Tr}\left[\left(3X\otimes X+I\otimes I\right)\rho\right]=\frac{1}{4}-\frac{3}{4}V_2$, and the results in the main text are from the values of V_2 which are presented in Fig. 3a in the main text. For three photons, V_3 oscillates between the expectation value of $X\otimes X\otimes X$, which is not a member of the stabilizing group, and $Y\otimes Y\otimes X$ which is a stabilizer. The three photon witness bound is $\langle\tilde{\mathcal{W}}_3\rangle=\frac{1}{2}-\frac{1}{8}\mathrm{Tr}\left[\left(7Y\otimes Y\otimes X+I^{\otimes 3}\right)\rho\right]=\frac{3}{8}-\frac{7}{8}V_3$, and the results in the main text are from the values of V_3 which are presented in Fig. 3b in the main text.

For four photons, V_4 oscillations include contributions from only the $Y\otimes X\otimes Y\otimes X$ stabilizer, which is enough in principle to bound all other stabilizers. In addition, we do measure another stabilizer directly, as $V_{4'}=\langle X\otimes I\otimes X\otimes X\rangle$. The I operator is equivalent to a partial trace over the second photon, effectively interfering contributions from only three photons. Thus, this second value is used to bound all stabilizers that include three or less Pauli operators. The four photon witness bound is $\langle\tilde{\mathcal{W}}_4\rangle=\frac{1}{2}-\frac{1}{16}\mathrm{Tr}\left[\left(5Y\otimes X\otimes Y\otimes X+10X\otimes I\otimes X\otimes X+I^{\otimes 4}\right)\rho\right]=\frac{7}{16}-\frac{5}{16}V_4-\frac{10}{16}V_{4'}$. The results in the main text are from the value of V_4 which is presented in Supplementary Figure 2 and $V_{4'}$, presented in Fig. 3c in the main text.

SUPPLEMENTARY NOTE 6 - QUANTUM INTERFERENCE LEVEL AND INDISTINGUISHABILITY

A requirement for perfect two-photon interference level $V_2 = 1$ is obtained when the two interfering photons are indistinguishable $M = 1$, and only one photon arrives at each pulse $g^{(2)}(0) = 0$. We relate these two terms in a similar manner to the derivation in [3] (appendix A.1). As was previously derived in [4], the indistinguishability arises from the mean-wave packet overlap

$$M = \mathrm{Tr}\left(\hat{\rho}_i\hat{\rho}_j\right) = \iint_{-\infty}^{+\infty} d\omega_i d\omega_j f\left(\omega_i\right) g\left(\omega_j\right) \left|\langle\omega_i|\omega_j\rangle\right|^2. \tag{22}$$

In an Hong-Ou-Mandel (HOM) experiment [5] the coincidence probability (P_{cc}) dip depends on the indistinguishability of two photons from the QD source, when assuming a perfectly balanced beam-splitter (BS) and $g^{(2)}(0) = 0$

$$P_{\mathrm{cc}}\left(\delta\tau\right) = \frac{1}{2}\left(1 - M\left(\delta\tau\right)\right), \tag{23}$$

and the measured dip visibility is defined as $V_{HOM}=1-\frac{P_{\mathrm{cc}}(0)}{P_{\mathrm{cc}}(\infty)}=M$, where $P_{cc}(\infty)$ is the two-photon probability when the single-photons do not arrive simultaneously to the beam-splitter. When assuming there is a probability of

two-photon emission from the source $g^{(2)}(0) > 0$, a lower bound to the actual indistinguishability is given by:

$$M = V_{HOM} + g^{(2)}(0). \tag{24}$$

We relate this to the measured level of interference V_2 by the fiber system entangling PBS1 gate followed by the analyzing apparatus [6]. When two photons arrive as described to the entangling PBS1, assuming a perfectly balanced PBS and no polarization error, the outcome state is

$$\hat{\rho} = M\hat{\rho}_{\mathrm{id}} + (1-M)\hat{\rho}_{\mathrm{d}}, \tag{25}$$

where $\hat{\rho}_{\mathrm{id}}$ ($\hat{\rho}_{\mathrm{d}}$) is the the entangled (decohered) state. When performing a measurement of the level of interference for the observable $X\otimes X$ on both photons, $\hat{\rho}_{\mathrm{id}}$ would attribute to the traced matrix diagonal elements only two terms $\hat{\rho}_{\mathrm{id}}^{1,1}=\hat{\rho}_{\mathrm{id}}^{4,4}=\frac{1}{2}M$. While $\hat{\rho}_{\mathrm{d}}$ would equally contribute all terms due to the lack of coherence $\hat{\rho}_{\mathrm{d}}^{i,i}=\frac{1}{4}(1-M)$. Thus, the resulting outcome of the interference level measurement is

$$V_2 = 2(\frac{1}{2}M + \frac{1}{4}(1-M)) - 2\frac{1}{4}(1-M) = M. \tag{26}$$

Yet, when taking into account the probability of two-photon arriving in the same pulse ($g^{(2)}(0) > 0$), additional reduction in the measured interference level is present. In the following, we derive the probability of an unwanted two-photon event. There is a 25% probability that both photons, coming from the same pulse, enter the loop. Then, there is a 50% probability that these two photons would not exit the loop at the same time, meaning half the probability of a correct event. Thus, their projected state is $|h_1 v_2\rangle$ and the measurement along $X\otimes X$ would yield an equal contribution of all the measured diagonal terms of the density matrix. In such case, the density matrix reads:

$$\hat{\rho} = \left(1 - \frac{1}{2}g^{(2)}(0)\right)(M\hat{\rho}_{\mathrm{id}} + (1-M)\hat{\rho}_{\mathrm{d}}) + \frac{1}{2}g^{(2)}(0)\hat{\rho}_{\mathrm{2ph}}. \tag{27}$$

Both the indistinguishability and the two photon emission affect the resulting interference level

$$V_2 = \left(1 - \frac{1}{2}g^{(2)}(0)\right)M. \tag{28}$$

We defined two quantum interference level measures in the previous section, V_n for any n and $V_{n'}$ for even n. We generalise now the description of these M-dependent measures for higher photon number cluster states.

We first model the n-photon cluster generation using the PBS entangling gate. For n-photon generation, a two-photon post-selection process of the entangling PBS is repeated on the incoming new photon and the photon remaining in the loop $\hat{\rho}_0^{(n)}=\hat{\rho}^{(n-1)}\otimes\hat{\rho}_{\mathrm{p}}$

$$\epsilon(\hat{\rho}^{(n)}) = M\varepsilon_0\hat{\rho}_0^{(n)}\varepsilon_0 + (1-M)\frac{1}{2}\left(\varepsilon_0\hat{\rho}_0^{(n)}\varepsilon_0 + \varepsilon_1\hat{\rho}_0^{(n)}\varepsilon_1\right), \tag{29}$$

where $\varepsilon_0=I^{\otimes(n-2)}\otimes(I\otimes I+Z\otimes Z)$ and $\varepsilon_1=I^{\otimes(n-2)}\otimes(I\otimes Z+Z\otimes I)$.

Reordering this process, we may regard two contributions which are dependent on M. For the two-photon case $\varepsilon_0\hat{\rho}_0^{(2)}\varepsilon_0$ accounts for $\frac{1+M}{2}$, while $\varepsilon_1\hat{\rho}_0^{(2)}\varepsilon_1$ accounts for $\frac{1-M}{2}$. As resulted in Supplementary Eq. 26 the interference level measurement $V_2=\frac{1+M}{2}-\frac{1-M}{2}=M$. When performing the $X^{\otimes 3}$ measurement on $\hat{\rho}^{(3)}$ we are again considering the process on two terms that act on $\hat{\rho}^{(2)}\otimes\hat{\rho}_p$ thus $V_3=M\left(\frac{1+M}{2}-\frac{1-M}{2}\right)=M^2$. This is recurring to any n, $V_n=M^{n-2}\left(\frac{1+M}{2}-\frac{1-M}{2}\right)=M^{n-1}$. When considering V_n', we are performing the measurement when n is even. For n=4 the $X\otimes X$ part of the measurement would give the M dependence, and the $X\otimes I$ would give $\frac{1+M}{2}-\frac{1-M}{2}=M$, so $V_{4'}=M^2$. Thus, the dependence of the maximum amplitude of V_n on the distinguishability is M^{n-1}, while $V_{n'}$ scales as $M^{\frac{n}{2}}$.

Several quantum dot sources were used for the present measurements. We show in Supplementary Figure 3 two indistinguishability measurements. The indistinguishability histogram is measured in a path-unbalanced Mach-Zehnder interferometer. One of the arms has an optical length $k\times\Delta t$ longer than the other, k being an integer number and Δt the temporal distance between consecutive pulses. Thus, allowing to interfere two successively emitted photons on the second fiber beam-splitter of the interferometer. At the output of this fiber beam-splitter we place two single-photon detectors to carry out a time-correlated two-photon coincidence detection. The simultaneous coincidence counts at Δt=0 allow to quantify the degree of photon indistinguishability between the two interfering photons. Supplementary Figure 3 demonstrates this for a 12 ns delay.

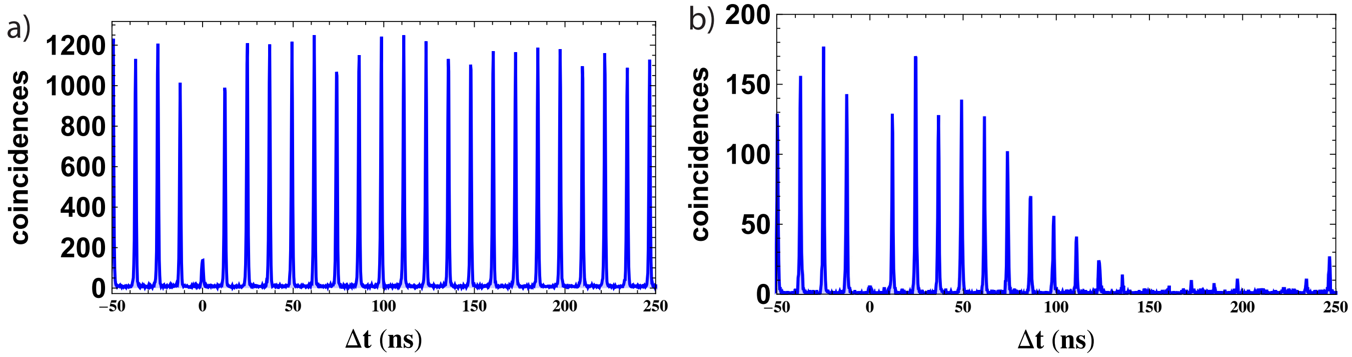

Supplementary Figure 3. **Example of two-photon indistinguishability measurement.** For photons delayed by 12 ns; the measure in panel (a)/(b) is performed under non-modulation/modulation of the train of laser pulses using charged-exciton sources without/with spectral filtering of the single-photon emission.

It is important to remark that the different pulse sequences used for the generation of different photon cluster state sizes (n=$2, 3, 4$ photons, see Fig. 3 of the main text) involve trains of emitted single photons separated by 74 ns. Therefore, the photon indistinguishability involved in the generation of cluster states is slightly lower than the one extracted in Supplementary Figure 3, due to QD spectral wandering [7].

Panel (a) corresponds to a negatively charged exciton which showed higher brightness. Such a source was used for the four-photon experiment. Without any spectral filtering, the measured M amounts to M=0.78±0.01. Note that this indistinguishability is substantially lower than the one observed on both neutral and positively charge excitons, indicating nuclear spin induced dephasing. The measurement shown in panel (b) was performed on positively charged exciton using a 10 pm spectral filtering to reduce the phonon sideband contribution resulting in an indistinguishability of M=0.95±0.01. The latter is taken with the pulse-modulation turned on, leading to the triangular envelope shape of the coincidence histogram.

The presence of an electron or a hole in the quantum dot is controlled following the method described in [8]. Maximum occupancy is obtained by finely adjusting the power and wavelength of a continuous wave non-resonant laser with wavelength typically between 890 nm and 905 nm.

SUPPLEMENTARY NOTE 7 - BACKGROUND COUNTS

The background correction was implemented by subtracting background measurement counts N^{bg} from the measured coincidence counts (CC) N^{meas}. Phase scans were performed acquiring CC data, N_i^{meas}, i=$1..2^n$ according to the desired pulse sequence - $\underbrace{11...1}_{n}00$, where n is the number of passing pulses, corresponding to the length of the desired chain. As the phase scan completes, it is repeated in the form of background measurements, $n-1$ pulses are open meaning no CC of an n-photon state should be recorded. As there are n combinations corresponding to these background measurements $1^{(1)}...0^{(k)}...1^{(n)}00$, this process is repeated for each combination with k=$1..n$, recording the background CC $N_{i,k}^{\mathrm{bg}}$. The final outcome N_i, which is the full pulse signal CC subtracting the background counts, is thus

$$N_i = N_i^{\mathrm{meas}} - \sum_{k=1}^{n} N_{i,k}^{\mathrm{bg}}. \tag{30}$$

The source of the background counts is from photons constantly entering the fiber system with no synchronization to the EOM pulse sequence. This is partly due to the EOM extinction ratio (1:100), which amounts to at most 1% of the coincidences. Here, the majority of the background signal is due to a residual single photon emission arising from the non-resonant laser pump used to insert a charge in the quantum dot. It amounts to typically ∼10% of the coincidences for a coincidence window of 5 ns. In a future experiment, this technical issue could be addressed in different ways, either by modulating the non-resonant pump laser, or using a smaller coincidence window.

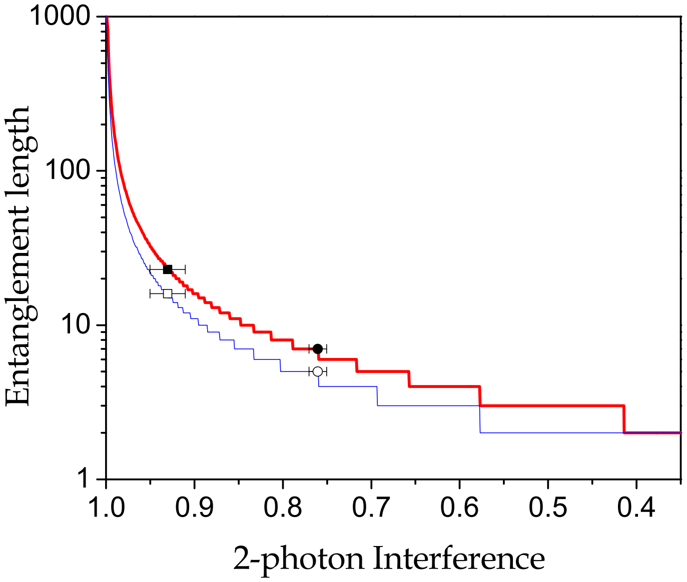

Supplementary Figure 4. **Entanglement length (EL) dependence on the two-photon interference.** The red thick and blue thin plots represent the calculation for distinguishing and depolarizing noise, respectively. For the measured two-photon interference levels of 0.93±0.02 and 0.76±0.01 the corresponding entanglement length is 23 (solid square) and 7 (solid circle) for the distinguishing noise and 16 (open square) and 5 (open circle) for the depolarizing noise. Errors are calculated from fitting to experimental data.

SUPPLEMENTARY NOTE 8 - ENTANGLEMENT LENGTH

The entanglement length measure $\mathcal{L}$ was introduced and analyzed in [3]. It is defined as the longest possible linear cluster state that will result with a pair of photons with positive concurrence after all photons, but the first and last of the chain, are measured along the $\hat{y}$ axis. This length is an upper limit to how far can quantum information flow along the linear state during the one-way quantum computation procedure [9]. The main noise factor taken is the indistinguishability of photons, modeled by the number of modes N_{m}. This corresponds to the two-photon quantum interference level in the diagonal basis $N_{\mathrm{m}}=\frac{1}{V_2}$. For n-photon creation, the process is detailed in Supplementary Eq. 29.

In this work we add the possibility of a general "white noise" as the source of noise in the process.

It is of interest to evaluate the presence of genuine multi-party entanglement in the created states, and extrapolate the system potential if time was not a practical constraint. For that purpose we use the entanglement length measure. Supplementary Figure 4 presents the dependence of this entanglement length on the measured two-photon interference level, assuming two different noise sources. In our experiment, the lack of perfect indistinguishability between consecutively emitted photons accounts for most of the reduced interference level. Calculating the entanglement length under this noise source results with the red thick line. It accounts for the possibility of a genuinely entangled linear state of 7-23 photons depending on the source M factor. The two-photon interference level can also be inferred directly from the interference levels of states of more photons. The measured four-photon interference level implies a state of 6 genuinely entangled photons as well. Moreover, when considering the worst case scenario of a uniformly depolarizing noise source, it provides a lower limit for the entanglement length for a measured two-photon interference level. In case of such a noise source, the dependence on the photon source V_2 factor will result in 5-16 entangled photons. Thus, we have shown that regardless of the applied noise model, the presented four photon linear cluster state is genuinely entangled.

When regarding V_2 to originate from some general noise process, similarly to the dependence derived from Supplementary Eq. 29, we find it dependence $V_2=(1-\delta)$, where δ is the amount of noise introduced - for the case of indistinguishability noise $M=1-\delta$. Again we can generalize this to any n, so $V_n=(1-\delta)^{n-1}=V_2^{n-1}$.

For a certain linear cluster state, that could be represented by a chain of length n, we may set a threshold value to the minimal value of V_2 for which the entanglement length is the chain length, $\mathcal{L}_{\min V_2}(n)=n$. From observing the solid blue thin line in Supplementary Figure 4, the calculation gives a value of V_2 that satisfies the threshold condition. For $n=2$ it is clear that $\min V_2=\frac{1}{3}$. For any added photon in the chain, this factor increases, as it is the minimal value between two photons before the removal of one from the chain. Thus, for any number of n, $\min V_2=\left(\frac{1}{3}\right)^{\frac{1}{n-1}}$. Meaning,

the larger the state is, the higher the two-photon quantum interference level should be to sustain the entanglement length.

We can further determine a threshold for the minimal n-photon quantum interference level $\min V_n$. From the previous relations we come to $\min V_n = \min V_2^{n-1} = \frac{1}{3}$ for any n.

SUPPLEMENTARY NOTE 9 - PARAMETRIC DOWN-CONVERSION SCALING RATIO

We start with the state definition of an n photon pair in mode a and b produced in the Parametric Down-Conversion (PDC) process

$$|\psi\rangle = \sqrt{1-|\lambda|^2}\sum_{n=0}^{\infty} |\lambda|^n |n\rangle_{\rm a}|n\rangle_{\rm b}, \tag{31}$$

this is a two-mode squeezed vacuum with strong quantum correlations [10]. λ is the squeezing parameter with its absolute square proportional to the pump power. For this derivation we assume non photon-number discriminating detectors ('bucket') with 100% efficiency. The probability for a single pair emission $|1\rangle_{\rm a}|1\rangle_{\rm b}$ is $P^{(1)}(1)=(1-|\lambda|^2)|\lambda|^2$. The rate for N consecutive photon pairs emission is then $\left(P^{(1)}(1)\right)^N = \left((1-|\lambda|^2)|\lambda|^2\right)^N$. When using this source as a single photon source in our scheme, each pair contributes one photon to the entangled state (the other photon being the heralded). Yet, as we are not employing photon-number-resolving detectors any $|k\rangle_{\rm a}|k\rangle_{\rm b}$, $1 \le k \le n$ per pulse would be considered as a single pair emission. Thus, the probability for a detection in modes a and b is

$$P^{(n)}(1) = 1 - P(0) = 1 - (1-|\lambda|^2) = |\lambda|^2. \tag{32}$$

The rate for N consecutive photon pairs emission is then $R_N = \left(P^{(n)}(1)\right)^N = \left(|\lambda|^2\right)^N$. The scaling ratio is then the probability of adding another pair

$$r = \frac{R_N}{R_{N+1}} = \frac{1}{|\lambda|^2}. \tag{33}$$

From the description of the interaction Hamiltonian of PDC $\lambda = tanh(\tau)$, where the interaction parameter τ is a linear function of the non-linear crystal properties and the pump electrical field strength, meaning an increase in r reflected in the brightness is achievable by increasing one of the parameters τ depends on [11].

We now turn to examine the two-photon quantum interference level dependence on τ [11, 12]. As presented in Fig. 4b in the main text, we assume only physical considerations, meaning perfect detection efficiency, perfect optical and polarization components and perfect indistinguishability between photons. In the PDC apparatus employing the photon's polarization degree of freedom, the quantum interference level is defined using a polarization analyzing system followed by 4 single photon detectors. Coincidence counts are recorded and the quantum interference level is defined as

$$V_2 = \frac{P_{14}+P_{23}-P_{13}-P_{24}}{P_{14}+P_{23}+P_{13}+P_{24}}. \tag{34}$$

From this definition we are left with a relation between the V_2 and τ

$$V_2 = \frac{1-\tanh^2\tau}{1+\tanh^2\tau}. \tag{35}$$

We are now able to relate the scaling ratio dependence on the interaction parameter

$$r = \frac{1+V_2}{1-V_2}, \tag{36}$$

we may notice that an increase in r would come at a cost of reduced quantum interference level. Yet, for the entangling process using parametric processes as heralded single photon sources the quantum interference level is [13]

$$V_2 = \frac{4}{-1+4\cosh(2\tau)+\cosh(4\tau)}. \tag{37}$$

Relating this to r gives

$$V_2 = \frac{(1-r)^2}{r(2+r)-1}, \tag{38}$$

This relation is displayed as the solid line in Fig. 4b in the main text.

SUPPLEMENTARY NOTE 10 - ANCILLARY DETERMINISTIC GATE EFFICIENCY

It is possible to compare our proposed scheme to the deterministic gate approach which relies on ancillary photons and linear optics [14]. In the deterministic approach $6.5n$ Bell pairs are required to ensure the generation of an n-photon cluster state. This scheme has a potential scaling ratio of r=1 that surpasses our scheme which relies on a 50% successful entangling operation, thus potentially reaching r=2. However, it is also possible to derive a comparison to our proposed method relying on the total efficiency.

Assuming the same total creation and detection efficiency $\eta = \frac{1}{r}$ for both methods. In our loop entangling method where a 50% chance of success gate is employed, the n-photon detection efficiency is $\eta_n^{(l)} = \left(\frac{1}{2}\right)^{n-1} \eta^n$. On the other hand, for the deterministic method, where 13 photons are required to be detected for a single qubit, the n-photon detection efficiency is $\eta_n^{(d)} = \eta^{13n}$.

Defining a threshold efficiency η_{th} for which $\eta_n^{(d)} \geq \eta_n^{(l)}$ gives

$$\eta_{\mathrm{th}} \geq \left(\frac{1}{2}\right)^{\frac{n-1}{12n}} \tag{39}$$

This threshold is saturated for large n, and so necessarily $\eta_{\mathrm{th}} \geq 0.944$, which could in turn be presented by the scaling ratio limited to $1 \leq r \leq 1.059$, for which the determinstic gate approach is necessarily superior.

SUPPLEMENTARY REFERENCES

[1] Raussendorf, R., Browne, D. E. & Briegel, H. J. Measurement-based quantum computation on cluster states. *Phys. Rev. A.* **68,** 022312 (2003).

[2] Pilnyak, Y. et al. Multi-qubit stabilizer states witness bounds from one measurement setting using phase measurements. *In preparation* (2020).

[3] Pilnyak, Y. et al. Simple source for large linear cluster photonic states. *Phys. Rev. A* **95,** 022304 (2017).

[4] Sun, F. W. & Wong, C. W. Indistinguishability of independent single photons. *Phys, Rev. A* **79,** 013824 (2009).

[5] Hong, C. K., Ou, Z. Y. & Mandel, L. Measurement of subpicosecond time intervals between two photons by interference. *Phys. Rev. Lett.* **59,** 2044–2046 (1987).

[6] Mandel, L. Coherence and indistinguishability. *Opt. Lett.* **16,** 1882–1883 (1991).

[7] Loredo, J. C. et al. Scalable performance in solid-state single-photon sources. *Optica* **3,** 433–440 (2016).

[8] Hilaire P. et al., Deterministic assembly of a charged quantum dot-pillar cavity device. Preprint at http://arxiv.org/abs/1909.02440 (2019).

[9] Raussendorf, R. & Briegel, H. J. A one-way quantum computer. *Phys. Rev. Lett.* **86,** 5188–5191 (2001).

[10] Kok, P. et al. Linear optical quantum computing with photonic qubits. *Rev. Mod. Phys.* **79,** 135–174 (2007).

[11] Eisenberg, H. S., Khoury, G., Durkin, G. A., Simon, C. & Bouwmeester, D. Quantum entanglement of a large number of photons. *Phys. Rev. Lett.* **93,** 193901 (2004).

[12] Takeoka, M., Jin, R.-B. & Sasaki, M. Full analysis of multi-photon pair effects in spontaneous parametric down conversion based photonic quantum information processing. *New. J. Phys.* **17,** 043030 (2015).

[13] Pilnyak, Y., & Eisenberg, H. S. Scalability and entanglement of multi-photon states with single-photon detectors. *In preparation* (2020).

[14] Browne, D. E. & Rudolph, T. Resource-efficient linear optical quantum computation. *Phys. Rev. Lett.* **95,** 010501 (2005).
